# Gut–lung axis and microbiome alterations in mycobacterial infections: from pathogenesis to therapeutic potential

**DOI:** 10.1080/19490976.2025.2612428

**Published:** 2026-01-07

**Authors:** Kimin Kang, Joong-Yub Kim, Jae-Joon Yim, Donghyun Kim

**Affiliations:** a Department of Biomedical Sciences, Seoul National University College of Medicine, Seoul, Republic of Korea; b Biomedical Research Institute, Seoul National University Hospital, Seoul, Republic of Korea; c Department of Microbiology, Harvard Medical School, Boston, MA, USA; d Division of Pulmonary and Critical Care Medicine, Department of Internal Medicine, Seoul National University Hospital, Seoul National University College of Medicine, Seoul, Republic of Korea; e Department of Microbiology and Immunology, Seoul National University College of Medicine, Seoul, Republic of Korea; f Institute of Infectious Diseases, Seoul National University College of Medicine, Seoul, Republic of Korea; g Institute of Cancer Research, Seoul National University, Seoul, Republic of Korea

**Keywords:** Gut–lung axis, microbiome, tuberculosis, non-tuberculous mycobacteria, dysbiosis, short-chain fatty acids, precision medicine

## Abstract

Mycobacterial lung diseases, including tuberculosis (TB) and nontuberculous mycobacterial pulmonary disease (NTM-PD), are increasingly recognized as disorders influenced not only by host immunity but also by microbiota. Emerging evidence identifies the gut–lung axis (GLA) as a key bidirectional communication network linking intestinal and pulmonary homeostasis. Mycobacterial infection itself induces airway and gut dysbiosis through immune and metabolic disturbances, which is further exacerbated by prolonged antibiotic therapy. Dysbiosis within either site reciprocally affects the other via GLA, leading to reduced microbial diversity, impaired epithelial integrity, and systemic inflammation. These alterations disrupt metabolite-mediated immunoregulation and attenuate IL-22–driven epithelial defense, thereby weakening bacterial clearance and promoting chronic inflammation. Distinct microbial features, such as the depletion of beneficial SCFA-producing taxa and enrichment of pro-inflammatory anaerobes, are observed in both TB and NTM-PD. Moreover, therapy-induced microbiome remodeling influences treatment response and disease relapse. Restoring microbial balance through probiotics, prebiotics, postbiotics, dietary modulation, or fecal microbiota transplantation offers a promising adjunctive strategy. This review integrates current evidence linking microbiome dysbiosis to mycobacterial pathogenesis and highlights microbiome-targeted interventions as an emerging therapeutic frontier in pulmonary mycobacterial diseases.

## 1. Introduction

### 1.1. Epidemiological and pathobiological overview of mycobacterial lung diseases

Mycobacteria constitute a genus within the *Actinobacteria* phylum, distinguished by their diverse pathogenic and environmental roles.^1^^,^^2^ Among them, *Mycobacterium tuberculosis* complex (MTB), the causative agent of tuberculosis (TB), has long been recognized as one of the world’s most important infectious pathogens. Despite declining incidence in most high-income countries, it remains endemic in sub-Saharan Africa, South Asia, and parts of Eastern Europe.^3^ MTB predominantly infects humans, and transmission occurs through aerosolized droplets.^4^^,^^5^ Even with substantial progress in global control efforts, millions of new TB cases continue to emerge each year, demonstrating the persistent public health burden of the disease.^6^

In parallel, more than 200 species of nontuberculous mycobacteria (NTM), mycobacteria other than the *M. leprae* and MTB, have been identified. ^7^ Historically considered saprophytic organisms, they are now recognized as significant opportunistic pathogens, particularly in immunocompromised individuals and older adults with pre-existing chronic lung disease.^7^^,^^8^ NTM pulmonary disease (NTM-PD) typically occurs in individuals with pre-existing structural lung abnormalities, such as bronchiectasis, chronic obstructive pulmonary disease (COPD), or prior TB-related lung damage, which create an environment that is permissive to colonization by opportunistic microorganisms including NTM.^9^^,^^10^ The prevalence of NTM infections has been rising in high-income regions, partly due to improved diagnostic tools and greater clinical awareness, but also due to aging population, increased life span of susceptible individuals, and greater environmental exposure.^11‐14^ Unlike TB, NTM-PD is not typically transmitted from person to person, but it presents a growing clinical challenge due to diagnostic complexity, species-dependent variability, and suboptimal treatment outcomes.^15^^,^^16^ Despite extensive research into the intricate pathogenesis of both TB and NTM diseases, neither disease can be fully explained by pathogen virulence alone or by host immunity in isolation, highlighting the importance of additional modulatory factors such as the microbiome.

### 1.2. The gut–lung axis: a bidirectional microbial–immune network

Although anatomically distinct, the gut and lung share comparable mucosal immune architectures and are interconnected through systemic circulation and immune cell trafficking. The gut–lung axis (GLA) refers to a complex communication network integrating microbial, metabolic, and immune signals that link the intestinal and respiratory systems. Beyond local effects of microbiota through direct interactions with respective mucosal immune systems, gut microbiota influences respiratory diseases via the GLA.^17–19^ Signals originating in the gut can influence pulmonary immunity via several mechanisms. First, microbial metabolites such as short-chain fatty acids (SCFAs) enter the circulation and modulate immune responses in the lung by promoting regulatory T (Treg) cell differentiation, suppressing pro-inflammatory cytokines, and enhancing epithelial barrier integrity.^20^ Second, gut-resident dendritic cells can traffic to secondary lymphoid organs, where they shape systemic T cell and B cell responses that ultimately affect the respiratory mucosa.^21‐23^ Third, microbial products such as lipopolysaccharides can translocate across a compromised intestinal barrier, triggering systemic inflammation that impacts lung homeostasis.^24^^,^^25^

Perturbations in the gut microbiome, commonly referred to as gut dysbiosis, have been increasingly associated with a broad range of respiratory disorders, including chronic infections caused by MTB and NTM, acute respiratory infections caused by influenza virus, *Streptococcus pneumoniae*, and *Pseudomonas aeruginosa*, and even chronic lung diseases such as asthma, COPD, and cystic fibrosis.^26‐32^ Experimental studies in murine models further support a causal link between gut dysbiosis and respiratory vulnerability. Disruption of the gut microbial community, through antibiotic exposure or germ-free conditions, increases susceptibility to respiratory pathogens such as *S. pneumoniae*, *P. aeruginosa*, and MTB.^33‐36^ Conversely, inoculation with specific bacterial consortium (genera *Lachnospira*, *Veillonella*, *Faecalibacterium*, and *Rothia*, which constitute early-life microbiota markers of asthma risk) as well as fecal microbiota transplantation (FMT) has been shown to alleviate airway inflammation.^37^^,^^38^ These findings indicate that gut dysbiosis can profoundly shape pulmonary immune responses.

**Figure 1.**
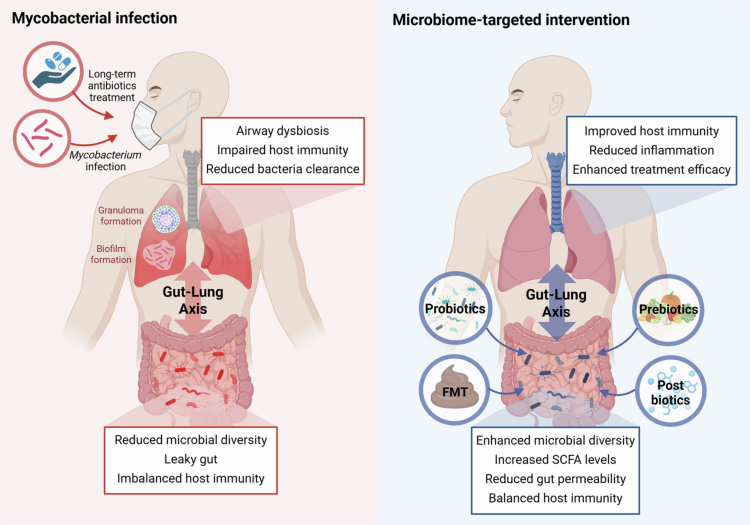
Overview of the gut–lung axis in mycobacterial infection and microbiome-based therapeutic strategies. (*Left*) Mycobacterial infection leads to granuloma and biofilm formation in the lung and also induces airway and intestinal dysbiosis through immune and metabolic disturbances. Long-term antibiotic treatment further exacerbates this imbalance. Through the gut–lung axis, dysbiosis in one compartment reciprocally affects the other, resulting in reduced microbial diversity, increased gut permeability, and impaired host immunity, which collectively lead to weakened bacterial clearance and chronic inflammation. (*Right*) In contrast, microbiome-targeted interventions such as probiotics, prebiotics, postbiotics, and fecal microbiota transplantation (FMT) restore gut–lung microbial crosstalk by enhancing microbial diversity and short-chain fatty acid (SCFA) production, strengthening epithelial barrier integrity, and rebalancing immune responses—ultimately improving host defense and treatment efficacy.

Notably, however, the relationship between the intestinal and respiratory systems is not unidirectional but bidirectional, with each influencing the other through immune and metabolic signaling.^26^^,^^39^ Just as gut microbial alterations can influence the pulmonary environment, lung infections and inflammation, including those occurring in the context of TB and NTM-PD, can in turn modify intestinal microbial composition through immune-mediated signaling and systemic inflammatory responses (Figure 1, *left*).^26^^,^^40^^,^^41^ For example, respiratory infections can increase gut permeability, promote bacterial translocation, and induce secondary dysbiosis (Figure 1, *left*).^42^ Such feedback loops highlight the integrative nature of mucosal immunity and reinforce the need to view the gut and lung as components of a unified microbial–immune system (Figure 1, *left*).

These observations reveal that the microbiota, interconnected through GLA, can play a pivotal role in regulating host immunity, disease susceptibility, and therapeutic response in mycobacterial infections (Figure 1, *left*). In line with this perspective, this review aims to appraise and integrate current evidence on how the microbiota influences mycobacterial pathogenesis and therapeutic responses through GLA. We further delineate shared and distinct microbiome alterations in TB and NTM-PD and discuss how these insights may guide the development of microbiome-modulating personalized therapeutic strategies.

## 2. Divergent pathogenesis in TB vs NTM-PD and host immune responses

### 2.1. Immune evasion and persistence mechanisms of MTB

MTB is a highly adapted intracellular pathogen that primarily targets macrophages and has evolved sophisticated mechanisms to evade host immune defenses and establish long-term persistence.^43^ Following phagocytosis, MTB employs multiple strategies to resist phagosomal destruction, including the secretion of effector proteins such as SapM and PtpA, which inhibit phagosome maturation and acidification.^43‐45^ These mechanisms enable MTB to survive within a protected intracellular niche and constitute a critical foundation for long-term persistence and latent infection.

Moreover, this capacity for sustained intracellular survival is closely linked to the formation of granulomas, highly organized immune structures composed of a central core of infected macrophages and necrotic debris, surrounded by layers of epithelioid cells, foamy macrophages, and lymphocytes, which contribute to restriction of bacterial dissemination.^46^^,^^47^ Granulomas create a dynamic microenvironment in which mycobacteria may adopt a spectrum of metabolic states ranging from active replication to non-replicating persistence, thereby enabling both bacterial containment and long-term survival under immune pressure, with the potential for reactivation under immunosuppressive conditions (Figure 1, left).^47^^,^^48^ Thus, in TB, granuloma formation represents not only a host defense strategy but also a central component of MTB pathogenesis.

In addition to intracellular persistence and granuloma-associated latency, emerging evidence suggests that MTB may adopt a biofilm-like mode of growth in advanced disease settings, particularly within necrotizing granulomas and cavitary lesions.^49^ This biofilm-like phenotype further contributes to treatment refractoriness and chronic infection.

### 2.2. Immune evasion and persistence mechanisms of NTM

In contrast to the relatively conserved virulence strategies of MTB, NTM exhibit substantial interspecies and strain-level heterogeneity in their pathogenic mechanisms. Several NTM species alter surface glycopeptidolipids to inhibit macrophage activation and suppress TLR2-mediated signaling, thereby dampening innate immune recognition and promoting persistence.^50^^,^^51^ Many clinically relevant NTM species, including *M. avium* and *M. abscessus*, are also capable of intracellular survival within macrophages by delaying phagosome–lysosome fusion and disrupting phagosomal membrane integrity through species-specific virulence factors.^52^ In addition, they display a strong capacity to form biofilms composed of lipid-rich extracellular matrices containing mycolic acids, glycolipids, and polysaccharides, which has been shown to contribute significantly to antibiotic tolerance and bacterial persistence both *in vitro* and on airway surfaces *in vivo*.^53‐55^

Granulomatous inflammation is likewise a pathological hallmark of NTM lung disease.^56^ However, unlike in TB, the functional role of granulomas in long-term bacterial persistence remains less well defined in NTM infection. Moreover, convincing evidence for organized NTM biofilm architectures within granulomas is still limited, and the spatial and functional relationship between granuloma formation and NTM biofilms remains largely inferential. This raises the possibility that, unlike TB where granulomas represent the dominant persistence niche, NTM persistence may rely more heavily on biofilm-based survival strategies, although this hypothesis remains insufficiently tested *in vivo*. In any case, from a diagnostic perspective, it remains challenging to distinguish radiologically or histopathologically whether granulomatous lesions and associated biofilm-like structures are attributable to MTB or NTM.^57^^,^^58^

Despite growing insight into these pathogen-specific mechanisms, the molecular determinants governing mycobacterial persistence, immune evasion, and host adaptation remain incompletely understood and appear highly species-specific. Because pathogen-intrinsic virulence factors alone cannot fully explain the marked heterogeneity in disease progression observed in both TB and NTM lung disease, increasing attention has shifted toward host-associated factors that modulate immune control and persistence. Against this backdrop, accumulating evidence suggests that MTB- or NTM-associated dysbiosis may modulate the immune microenvironment in ways that influence granuloma-associated immune regulation, particularly through IL-17–dependent pathways, although causal relationships remain to be firmly established.^59‐61^

### 2.3. Divergent immune polarization and host susceptibility in TB and NTM disease

At the systemic level, the distinct immune evasion strategies of MTB and NTM culminate in fundamentally different patterns of T-cell polarization and host susceptibility. The gut microbiota plays a central role in shaping T-cell differentiation and immune homeostasis.^62^ For instance, *Bacteroides fragilis* produces polysaccharide A that modulates dendritic cell function through TLR2 signaling, leading to the induction of Treg cells and IL-10 production, thereby promoting immune homeostasis in the gut.^63^^,^^64^ Conversely, segmented filamentous bacteria stimulate epithelial serum amyloid A production, which drives IL-6 and IL-23 secretion by dendritic cells and promotes Th17 differentiation.^65^^,^^66^ Microbial metabolites such as short-chain fatty acids further contribute to immune homeostasis by inducing IL-22 production and supporting epithelial barrier integrity and Treg function.^20,67‐69^ Additionally, through GLA, the intestinal microbiota can shape pulmonary T cell populations and influence T cell–dependent immune responses that differentially affect MTB and NTM infections.^21^^,^^22^^,^^40^^,^^41^ However, the precise mechanisms by which microbiota-derived signals selectively modulate TB versus NTM susceptibility remain poorly defined.

In addition to microbiota-derived regulation of T cell subsets, mycobacterial infection per se likely exerts a critical influence on T cell subtype differentiation. MTB infection is classically associated with a dominant Th1 response (IFN-*γ*), while Th17 responses can also be induced, particularly in early or mucosal phases, depending on context. IFN-*γ* plays a central role in macrophage activation by promoting phagosome maturation, reactive nitrogen intermediate production, and antigen presentation.^70^^,^^71^ Th17 cells further contribute to protection through neutrophil recruitment and IL-22–mediated mucosal barrier maintenance, although excessive Th17 activity can exacerbate tissue pathology.^72‐74^ Thus, TB represents a tightly regulated balance between protective cellular immunity and immunopathology.

In contrast, NTM-PD occurs predominantly in individuals with structural airway abnormalities or impaired mucosal defenses, conditions frequently associated with Th2-skewed immune profiles. In some NTM-infected cohorts, elevated biomarkers of Th2 inflammation, such as increased eosinophil counts and higher total IgE have been observed, suggesting a shift toward humoral-type or non-protective immune polarization in susceptible individuals. Although a generalized Th2 bias across all NTM infections remains controversial, multiple studies suggest that Th2-prone conditions may predispose individuals to NTM colonization and persistence.^75^^,^^76^ Notably, several bacterial taxa enriched in NTM-PD, including *Veillonella* and *Prevotella*, have been linked to heightened Th17-driven pulmonary inflammation, suggesting that NTM-associated dysbiosis may promote pathogenic Th17 responses rather than effective bacterial clearance.^77^^,^^78^ Collectively, these findings suggest that NTM-PD may arise within heterogeneous immune environments, ranging from Th2-skewed, non-protective responses in structurally vulnerable hosts to dysbiosis-driven Th17-dominant inflammatory states that promote tissue damage without effective bacterial clearance.

It remains unclear whether MTB and NTM directly drive T cell polarization or whether this process is indirectly influenced by microbiota-associated mechanisms. Notably, the presence of certain commensal species, such as *Haemophilus*, has been associated with increased expression of Th1-related genes in the airways of TB patients.^79^ Moreover, murine studies demonstrate that early intestinal colonization with *Helicobacter hepaticus* promotes the accumulation of activated and senescent T-cell populations and increases susceptibility to MTB challenge.^80^

### 2.4. Knowledge gaps and therapeutic implications in microbiota–mycobacterial interactions

Despite growing insights into host–pathogen–microbiota interactions, significant knowledge gaps remain, particularly regarding NTMs. Unlike MTB, whose virulence mechanisms and immune correlates of protection are relatively well characterized, NTM species exhibit extreme heterogeneity in both pathogenic strategies and host immune responses. This diversity severely limits the generalizability of current mechanistic models and hampers the rational design of targeted immunomodulatory or microbiota-based interventions.

Furthermore, it remains unresolved whether microbiota alterations are a cause or a consequence of mycobacterial infection, and whether therapeutic manipulation of microbial communities can meaningfully alter disease trajectories. A deeper, species-resolved understanding of NTM-host interactions, combined with mechanistic dissection of gut–lung immune crosstalk, will be essential for the development of effective host-directed and microbiota-based therapies.

## 3. Microbiome across the gut–lung axis in mycobacterial pulmonary disease

### 3.1. Methodological and biological contexts of the lung–gut microbiome in mycobacterial disease

Microbiome profiling in TB and NTM-PD is greatly affected by sampling methods, but it has still produced meaningful biological insights into mycobacterial lung disease. Sputum is easy to collect and widely used, but it may not accurately reflect the lower airway microbiome due to upper airway contamination; in contrast, bronchoalveolar lavage fluid (BALF) and protected specimen brushing (PSB) provide greater lower airway specificity but are semi-invasive, limiting repeated sampling. Lung tissue allows the most direct assessment of lung microbes, but it represents only small, localized areas of the airway and requires invasive procedure.^81^ Stool-based profiling is widely used for practical reasons, but fecal microbiota mainly reflects the colonic lumen, only indirectly representing lung–gut interactions, and is strongly influenced by diet and medications.^82^^,^^83^ Despite these constraints, converging evidence across independent cohorts supports a functionally relevant role of lung and gut microbiome dysbiosis in shaping host immunity and mycobacterial pathogenesis.

### 3.2. Airway microbiota in TB

Given that the airway microbiota can influence alveolar macrophage function, perturbations of this community may impair local immune crosstalk.^84^ Accordingly, the pulmonary microbiome has emerged as a crucial local modulator shaping disease severity and host–pathogen interactions in TB.

In the context of TB, many reports have demonstrated reduced *α*-diversity of the lung microbiome, indicating a narrower range of microbial species compared with healthy or MTB-uninfected individuals (Table 1). In parallel, *β*-diversity shows significant compositional differences between untreated TB patients and control groups (Table 1).^85‐87^ As noted above, despite substantial variability across studies driven by methodological and host-related factors, MTB infection has been associated with distinct airway microbial compositions compared with uninfected individuals (Table 1).^85‐87^ As anticipated, MTB was dominantly detected in untreated TB patients compared with uninfected control groups (Table 1).^85^^,^^86^ Beyond the presence of MTB itself, untreated TB patients also exhibit enrichment of pathogenic taxa. For example, *Staphylococcus aureus* is significantly enriched compared with healthy and lung cancer groups, a pattern that may be associated with increased susceptibility to secondary infections and worsened pulmonary outcomes. Conversely, commensal or potentially beneficial taxa, such as *Ralstonia pickettii*, which can activate ethionamide into its anti-mycobacterial form, and *Prevotella melaninogenica*, which has been associated with better lung function, are depleted in untreated TB patients (Table 1).^87^ Furthermore, *Streptococcus* dominance has been associated with a simplified airway microbiome of untreated active TB patients, accompanied by the displacement of anaerobic genera such as *Selenomonas* and *Fusobacterium* as community imbalance progresses (Table 1).^88^ Collectively, these findings suggest that airway microbiome dysbiosis in TB is associated with altered immune–microbial interactions in the lung, which may influence inflammatory states and disease manifestations, although causal relationships remain to be fully established.

**Table 1.** Airway and gut microbiota in MTB infection.

Sample; Analysis method	Study population	Microbiome diversity	Characteristic shifts in microbial composition	Other notes	Reference
BALF; Shotgun metagenomics	Suspected TB patients: MTB + (*n* = 30) vs. MTB– (*n* = 30)	Alpha diversity decreases in MTB + group compared to MTB– group. Beta diversity shows a significant distinction between MTB + and MTB–.	MTB + samples are dominated by MTB; MTB– samples are enriched with *Neisseria, Prevotella, Streptococcus*, *Selenomonas,* and *Bifidobacterium*. (Bacteria)	Fungal communities were analyzed by extracting sequencing reads taxonomically assigned to fungi from the shotgun metagenomic data	Hu et al. (2020)^85^
		Based on fungal read proportions, the MTB + group exhibited reduced fungal diversity compared to MTB–.	*Aspergillaceae* and *Malasseziaceae* are detected in all samples, while *Candida* is found only in MTB– samples*.* (Fungi)		
Sputum; 16S rRNA amplicon sequencing, ITS amplicon sequencing	Suspected TB patients: MTB + (*n* = 56) vs. MTB– (*n* = 41)	Chao1 index is significantly higher in MTB + group compared to MTB– group but Shannon index shows no significant difference. Beta diversity significantly divides each group. (Bacteria)	MTB + samples are enriched in MTB and *Stenotrophomonas maltophilia*; MTB– samples harbor higher *Prevotella melaninogenica*, *Veillonella parvula, Corynebacterium striatum,* and *Pseudomonas aeruginosa.* (Bacteria)	Correlation network analysis suggests that MTB shows both positive and negative associations with other microbes, consistent with potential cooperative and competitive relationships that may influence respiratory microbial community dynamics and TB disease progression.	Terbtothakun et al. (2025)^86^
		Alpha diversity shows no significant difference; Beta diversity differs significantly. (Fungi)	*Candida orthopsilosis* is enriched in MTB + samples, while *Aureobasidium leucospermi,* and *Wallemia muriae* are predominated in MTB– samples. (Fungi)		
BALF; Shotgun metagenomics	Untreated TB group (UTG, *n* = 12) vs. treated TB group (TTG, *n* = 15), cured TB group (CTG, *n* = 11), healthy control group (HCG, *n* = 8), lung cancer group (LCG, *n* = 7)	UTG shows significantly lower alpha diversity than HCG and LCG. Beta diversity shows significant compositional differences between UTG and both HCG and LCG. ; Anti-TB treated groups (TTG/CTG) show no significant alpha diversity difference between each group, but their alpha diversities are higher than UTG.	*Staphylococcus aureus* is enriched in UTG than HCG and LCG. HCG and LCG show higher abundances of *Prevotella melaninogenica* and *Ralstonia pickettii* than UTG.	Anti-TB treatment with first-line drugs increases the alpha diversity of the lung microbiota while concomitantly promoting the accumulation of antibiotic resistance genes. Anti-TB treatment leads to partial normalization of the lung microbiota rather than complete recovery.	Xiao et al. (2022)^87^
Sputum; 16S rRNA amplicon sequencing, Shotgun metagenomic sequencing	Untreated active TB patients (*n* = 334): Low burden vs. high burden, clinical severity	Microbiome diversity correlated with disease manifestations, including chest radiographic abnormalities, rather than showing clear case–control separation.	Disease severity was associated with Streptococcus dominance and a relative depletion of anaerobic genera such as *Selenomonas* and *Fusobacterium*.	An increasing inverse relationship between *Streptococcus* and anaerobic genera such as *Selenomonas* and *Fusobacterium* was associated with lower alpha diversity.	Ticlla et al. (2021)^88^
Feces; 16S rRNA amplicon sequencing, untargeted metabolomics	Untreated active TB patients (*n* = 83) vs. healthy controls (*n* = 52)	Alpha diversity is lower in TB patients compared with healthy controls. Beta diversities of metagenomics and metabolomics differ significantly between two groups.	*Bacteroidales, Prevotellaceae* and *Bacteroides vulgatus* are enriched and depleted SCFA-producing bacteria in TB patients compared with healthy controls. *Actinobacteria, Firmicutes, Clostridiales, Lachnospiraceae, Ruminococcaceae Bifidobacteriales* and *Blautia* are enriched in healthy controls compared with TB patients.	TB patients show reduced production of SCFAs and altered fecal metabolomic profiles compared with healthy controls.	Wang et al. (2022)^89^
Feces; Shotgun metagenomic sequencing	Untreated active TB patients (*n* = 30) vs. healthy controls (*n* = 31)	Alpha diversity is lower in TB patients than healthy controls. Beta diversity shows a significant difference between two groups.	SCFA-producing bacteria (*Bifidobacterium longum, Bifidobacterium adolescentis, Ruminococcus obeum,* and *Akkermansia muciniphila*) decrease in TB patients compared with healthy controls.	The healthy and diseased states are associated with distinct SNP patterns in *Bacteroides vulgatus*.	Hu et al. (2019)^90^
Feces; 16S rRNA amplicon sequencing, ITS amplicon sequencing	Untreated active TB patients (*n* = 33) vs. healthy controls (*n* = 20)	Alpha diversity decreases in TB patients compared with healthy controls for bacteria and fungi. Beta diversity separates TB patients from healthy controls.	*Bacteroides* and *Prevotella* increase but, *Blautia* and *Bifidobacterium* decrease in TB patients compared with healthy controls. (Bacteria)	A combined bacteria–fungi genus panel showed higher discriminatory performance (AUC = 0.996) than bacterial-only or fungal-only panels. Changes in key bacterial/fungal genera correlate with IFN-*γ* and IL-17 levels.	Han et al. (2024)^59^
			*Saccharomyces* increases, but *Aspergillus* decreases in TB patients compared with healthy controls. (Fungi)		
Feces; 16S rRNA amplicon sequencing	Untreated children with TB (*n* = 18) vs. healthy controls (*n* = 18); Before (*n* = 6) vs. after treatment (*n* = 6)	ACE, Chao1, and Shannon indices show no significant differences, but Simpson index decreases significantly in pediatric TB patients compared with healthy controls. No significant separation of samples is detectable between the two groups in beta diversity. ; The gut microbial richness in post-treated TB patients declines following one month of anti-TB treatment.	*Prevotella* (pro-inflammatory bacteria) and *Enterococcus* (opportunistic pathogen) are enriched, but beneficial bacteria (*Faecalibacterium prausnitzii, Ruminococcaceae, Bifidobacteriaceae*) are depleted in TB patients compared with healthy controls. ; No specific bacterial taxa differed significantly between pre- and post-treatment samples	Anti-TB treatment primarily reduces microbial richness without significantly altering taxonomic composition.	Li et al. (2019)^91^

Source: MTB: *Mycobacterium tuberculosis*; TB: tuberculosis; BALF: bronchoalveolar lavage fluid; MTB + : MTB infection was confirmed; MTB–: no evidence of MTB infection was detected; ITS: Internal Transcribed Spacer; SCFA: short-chain fatty acid; SNP: single nucleotide polymorphism.

### 3.3. Gut microbiota and the gut–lung axis in TB

Consistent with airway dysbiosis, TB patients also exhibit marked alterations in the gut microbiota. A reduction in gut microbial diversity is frequently observed compared with healthy controls (Figure 1, *left* and Table 1).^59^^,^^89^^,^^90^ The microbial alteration is characterized by markedly reduced levels of beneficial taxa such as *Bifidobacteriaceae, Ruminococcaceae,* and *Faecalibacterium prausnitzii* alongside an expansion of potential pathobionts such as *Enterococcus* relative to healthy individuals (Table 1).^91^ This dysbiosis often involves a decreased abundance of SCFA-producing taxa, including *Bifidobacterium adolescentis, B. longum, Ruminococcus obeum,* and *Akkermansia muciniphila*, which are predominant in healthy individuals (Table 1).^90^

These microbial shifts lead to a reduction in SCFA production, an essential factor for maintaining immune homeostasis and limiting inflammation (Figure 1, *left*).^92^ Importantly, clinical studies in TB patients have shown that butyrate induces cathelicidin-dependent MTB growth restriction and anti-inflammatory effects in macrophages, offering potential as a host-directed adjunctive therapy for TB.^93^ In addition, SCFAs, such as butyrate and propionate, promote Treg differentiation and suppress pro-inflammatory T cell responses in TB patients.^20^^,^^67^ Within the framework of the gut–lung axis, these observations support a functional link between gut dysbiosis and systemic immune modulation that may shape pulmonary responses during TB.

### 3.4. Airway microbiota in NTM-PD

Similar to observations in TB, alterations in airway microbial community structure have been observed in patients with NTM-PD, although changes in microbial diversity are heterogeneous across studies (Table 2).^78^^,^^94^^,^^95^ This dysbiosis has been associated with elevated inflammatory mediators in BALF in some studies, linking altered microbial communities to heightened local immune activation in the NTM-infected lung (Table 2).^78^ Notably, the pattern of airway dysbiosis in NTM-PD appears distinct from that observed in TB, suggesting disease-specific microbial restructuring. For instance, Yamasaki et al. reported that untreated NTM-positive non-cystic fibrosis bronchiectasis patients exhibit a distinct airway microbiome characterized by reduced *Haemophilus*, *Pseudomonas*, and *Staphylococcus*, but increased *Streptococcus*, *Prevotella*, and *Veillonella*, compared with NTM-uninfected patients (Table 2). This compositional shift may reflect microenvironmental changes, such as reduced oxygen availability, associated with NTM colonization.^95^ Caverly et al. reported that, in patients with cystic fibrosis, specific airway microbiome features preceding incident NTM infection, rather than NTM colonization per se, were associated with subsequent NTM pulmonary disease and persistent infection, including positive temporal associations with genera such as *Pseudomonas*, *Streptococcus*, *Veillonella*, *Prevotella*, and *Rothia* (Table 2).^96^ Across several cohorts, NTM-PD patients frequently exhibited increased levels of *Streptococcus*, *Veillonella*, and *Prevotella*, along with decreased *Staphylococcus*.^95^^,^^96^ Clinically, NTM-PD is classified into two major radiological forms: the fibrocavitary (FC) and nodular-bronchiectatic (NB) types, with the former characterized by a higher mycobacterial burden than the latter.^97^ Correspondingly, FC lesions are enriched in MTB along with genera such as *Faecalibacterium* and *Blautia*, whereas the NB form shows relative dominance of *Bacteroides* and *Clostridium* (Table 2).^98^ Rather than asking whether microbiome changes are a cause or a consequence of disease, these findings suggest that NTM-PD should be viewed as a dynamic process in which lung structure, microbial communities, and mycobacterial burden change together over time and collectively shape disease progression*.*

**Table 2.** Airway and gut microbiota in NTM infection.

Sample; Analysis method	Study population	Microbiome diversity	Characteristic shifts in microbial composition	Other notes	Reference
PSB, Bronchial washing; 16S rRNA amplicon sequencing	Untreated NTM-PD patients (*n* = 14) vs. Non-NTM-PD patients undergoing bronchoscopy (*n* = 10)	NTM-PD samples show lower trend of OTUs and Chao1 richness (non-significant) than controls. Beta diversity separates the two groups.	Abundances of *Rhodococcus, Pseudomonas, Cytophagaceae,* and *Alcaligenaceae* are higher in NTM-PD group compared with controls group.	PSBs and bronchial washings display similar patterns within each group but differ between the two groups. PSBs exhibit lower alpha diversity than bronchial washings, suggesting that PSB more selectively reflects the intrinsic microbiome of the lower airway.	Kang et al. (2021)^94^
Oral wash, Supraglottic sample, Sputum, BALF; 16S rRNA amplicon sequencing, Mycobacterium-enriched sequencing	Untreated bronchiectasis patients: NTM + (*n* = 61) vs. NTM– (*n* = 45)	No significant alpha or beta diversity differences between NTM + and NTM– in sputum/oral washing samples. ; BALF shows significant beta diversity difference across sample types including sputum.	NTM + BALF enriches *Mycobacterium*, *Oxalobacteraceae*; NTM– BALF enriches *Porphyromonas.*	Oral commensals (*Prevotella, Veillonella*, and *Leptotrichia*) are associated with increased neutrophils, IL-6, IL-17, IL-23 in NTM + BALF. Sputum microbiota resembles oral communities rather than lower-airway BALF.	Sulaiman et al. (2020)^78^
BALF; 16S rRNA gene clone library analysis*	Untreated bronchiectasis patients: NTM + (*n* = 29) vs. NTM– (*n* = 29)	*Microbial diversity could not be reliably assessed using this method.	Significantly higher anaerobe (*Prevotella*) rates in NTM patients. NTM + bronchiectasis shows reduced *Haemophilus*, *Pseudomonas, Staphylococcus* and enriched *Streptococcus, Prevotella, Veillonella.*	Collapse/consolidation are significantly associated with molecularly detected proportions of *Prevotella* species in BALF.	Yamasaki et al. (2015)^95^
Sputum; 16S rRNA amplicon sequencing	Untreated CF patients with NTM infection (*n* = 24; 188 samples): Before vs. after NTB infection, PD + vs. PD–, Persistent NTB infection (*n* = 8) vs. Transient NTM infection (*n* = 15)	No significant differences in alpha diversity were observed. Beta diversity differed significantly by NTM outcome, but not between persistent and transient infection.	Increases in *Prevotella, Streptococcus*, *Veillonella, Pseudomonas,* and *Rothia* are positively associated with the diagnosis of NTM-PD and persistent NTM infection.	Pre-NTM airway microbiota features in CF patients were associated with subsequent clinical outcomes, including NTM pulmonary disease and persistent infection.	Caverly et al. (2021)^96^
Lung tissue; 16S rRNA amplicon sequencing	NTM-PD patients undergoing surgical resection: Involved sites (FC cavity wall lesion vs. NB bronchiectasis lesion) vs. non-involved sites (grossly normal tissue), MAC (*n* = 16) vs. *M. abscessus* (*n* = 7)	Involved sites (FC and NB) in lung tissue show higher alpha diversity than non-involved tissue. Beta-diversity shows significant differences in overall lung microbiome between involved and non-involved lung tissues.; Beta-diversity differs significantly between lung tissues from patients with MAC-PD and those with *M. abscessus*-PD at involved sites.	Several genera including *Limnohabitans, Rahnella, Lachnospira, Flavobacterium, Megamonas, Gaiella, Subdoligranulum, Rheinheimera, Dorea, Collinsella*, and *Phascolarctobacterium* are enriched at involved sites, whereas *Acinetobacter* is increased at non-involved sites. ; At the involved sites, *Mycobacterium, Faecalibacterium*, *Bacteroides* and *Blautia* are enriched in FC lesions than in NB lesions. At non-involved sites, *Streptococcus* and *Escherichia* are significantly more abundant in the FC lesions than in NB lesions.	The higher mycobacterial burden observed in the FC form may be linked to more aggressive clinical outcomes compared with the NB form.	Kim et al. (2023)^98^
Feces, Sputum; 16S rRNA amplicon sequencing	NTM-PD patients (*n* = 10; *n* = 9, untreated & *n* = 1, at least 1 year after treatment completion) vs. healthy controls (*n* = 10); Low-BMI (*n* = 4) vs. Others (normal/high-BMI, *n* = 6)	Both sputum and fecal samples show reduced alpha diversity in NTM-PD compared to healthy controls. (feces: *p* < 0.001; sputum: trend) Beta diversity separates NTM-PD from controls in sputum and feces. Beta diversity significantly separated NTM-PD patients from healthy controls in both sputum and fecal samples and also differed significantly among the healthy, low-BMI, and others.	*Streptococcus constellatus, Sneathia sanguinegens, Treponema bryantii* and *Gemella morbillorum* are enriched in NTM-PD patients compared with controls.; Low-BMI exhibits higher abundances of several genera including *Actinotalea* and *Muribaculum* and enrichment of *Neisseria mucosa* and *Veillonella rodentium* compared with the others. (Sputum)	NTM-PD patients with lower BMI show reduced microbial diversity compared with those with higher BMI.	Choi et al. (2023)^99^
			*Blautia caecimuris, Tyzzerella nexilis, Clostridium innocuum*, *Enterocloster clostridioformis,* and *Erysipelatoclostridium ramosum* are enriched in NTM-PD patients compared with controls.; Low-BMI showed higher abundances of *Lachnoclostridium* and *Streptobacillus* at the genus level and enrichment of *Blautia wexlerae*, *Erysipelatoclostridium ramosum*, *Lachnoclostridium pacaense*, *Veillonella atypica*, and *Bacteroides thetaiotaomicron* compared with the others. (Feces)		
Feces; 16S rRNA amplicon sequencing	Untreated NTM-PD patients (*n* = 19) vs. healthy controls (*n* = 25) ; Antibiotics-treated mice model	In NTM-PD patients, alpha diversity significantly decreases compared with healthy controls. Beta diversity differs significantly between NTM-PD and healthy controls	*Prevotella_9, Megmonas* and *Veillonellaceae* are depleted, while *Bifidobacterium longum*, *Eubacterium hallii*, and *Clostridium* spp. are enriched in NTM-PD patients compared with healthy controls.	*Clostridium innocuum* group shows a positive correlation with disease severity, whereas *Prevotella_9* exhibits a negative correlation.; TLR2 ligand activity is reduced across feces, serum, and lung supernatants, accompanied by decreased TLR2 expression in the colon and lungs.	Lin et al. (2024)^27^
				*P. copri* or capsular polysaccharides can enhance TLR2 signaling and ameliorate NTM-PD susceptibility in mice.	

Source: NTM: nontuberculous mycobacteria; BALF: bronchoalveolar Lavage Fluid; PD: pulmonary disease; PSB: Protected specimen brushing, NTM + : NTM infection was confirmed; NTM–: no evidence of NTM infection was detected; CF: cystic fibrosis; MAC: *Mycobacterium avium* complex; BMI: body mass index; FC: fibrocavitary form; NB: nodular bronchiectatic form.

### 3.5. Gut microbiota and the gut–lung axis in NTM-PD

Patients with NTM-PD also exhibit significant gut microbial dysbiosis characterized by reduced microbial diversity (Table 2).^27^^,^^99^ *Blautia caecimuris*, *Enterocloster clostridioformis*, and *Clostridium innocuum* are more abundant in patients with NTM-PD than in healthy controls (Table 2).^99^ Among these, the *Clostridium innocuum* group, a potential opportunistic pathogen, correlates strongly with NTM-PD severity.^27^^,^^99^ In contrast, the abundance of *Prevotella*, particularly *Prevotella*_9, is inversely associated with disease severity (Table 2).^27^

Furthermore, administration of *Prevotella copri* or its capsular polysaccharides has been shown to enhance TLR2-mediated signaling and restore immune responsiveness, thereby ameliorating susceptibility to NTM-PD in experimental models (Table 2).^27^ By implicating gut-derived immune modulation in NTM-PD susceptibility, these findings highlight the gut microbiota as a potential target for host-directed interventions aimed at restoring immune balance along GLA.

### 3.6. Comparative microbiome features between TB and NTM-PD

From a comparative perspective, despite their etiological differences, both TB and NTM-PD exhibit marked dysbiosis at the respiratory and intestinal levels, characterized by a loss of microbial diversity, depletion of SCFA-producing commensals, and enrichment of pro-inflammatory or opportunistic pathobionts (Figure 1, *left*). These alterations contribute to immune dysregulation and disease persistence in both conditions.

Few studies have directly compared microbiome patterns between TB and NTM-PD. Belheouane et al. reported subtle yet meaningful differences in microbial diversity between TB and NTM-PD patients.^100^ TB patients display a slightly higher expected richness of amplicon sequence variants (ASVs) (Chao1 index, *p* = 0.07) than NTM-PD patients, while observed diversity (Shannon index) is comparable between groups (*p* = 0.14). At the ASV level, TB samples show enrichment of specific *Serratia* taxa (ASV_7 and ASV_21), which serve as discriminative markers between the two diseases.^100^ These findings suggest that modest variations in microbial composition may reflect distinct ecological conditions in TB- and NTM-affected lungs.

While the biological and clinical implications of these differences remain uncertain, they highlight the need for larger-scale and longitudinal studies to delineate disease-specific microbial trajectories and their functional relevance more precisely. Addressing this gap will be critical for advancing microbiome-informed diagnostics and therapeutic development.

### 3.7. Non-bacterial microbial dimensions in mycobacterial lung disease

In addition to bacterial communities, emerging evidence from TB studies indicates that fungal components of the lung and gut microbiome are also altered during mycobacterial infection, whereas comparable mycobiome data in NTM-PD remain largely unavailable.^59^^,^^86^^,^^101^

Shotgun metagenomic and ITS-based studies have begun to characterize alterations in the airway and gut mycobiome (Table 1). Analyzes of BALF and sputum samples indicate reduced fungal diversity in some cohorts of MTB-infected individuals compared with uninfected controls, accompanied by study-dependent shifts in fungal community composition.^59^^,^^86^ The clinical importance of airway fungi is further underscored by the high incidence of chronic pulmonary aspergillosis and other *Aspergillus*-related diseases following TB and in NTM-PD, linking fungal ecology to structural lung damage and long-term morbidity across mycobacterial lung diseases.^102‐104^ In parallel, analyzes of fecal samples from untreated TB patients relative to healthy controls reveal fungal changes that occur alongside bacterial dysbiosis and correlate with host immune markers such as IFN-*γ* and IL-17, suggesting immunomodulatory roles of the mycobiome in TB.^59^

Beyond fungi, gut archaea may influence host immune tone and metabolic homeostasis, potentially contributing to gut–lung axis regulation.^105^ However, direct evidence linking archaeal dysbiosis to mycobacterial lung disease remains limited. Collectively, these observations highlight that non-bacterial components of the microbiome represent an important but insufficiently characterized dimension of host–mycobacteria interactions, and thus require further integrated investigation, particularly through multi-kingdom and longitudinal approaches.

## 4. Microbiome-mediated variability in treatment outcomes

### 4.1. Microbiome-mediated variability in TB treatment outcomes

Variability in TB treatment outcomes cannot be fully explained by differences in disease severity, drug susceptibility, or patient adherence alone. Despite the use of standardized first-line regimens comprising isoniazid (INH), rifampicin (RIF), ethambutol (EMB), and pyrazinamide (PZA), substantial heterogeneity, ranging from delayed clinical improvement and persistent culture positivity to relapse, exists among patients.^106‐108^ Growing evidence suggests that this heterogeneity may be partly shaped by interindividual differences in the lung and gut microbiome, as well as by microbial perturbations during therapy (Table 3).^109‐112^

**Table 3.** Microbiome-mediated variability in mycobacterial disease treatment outcomes.

Sample	Study design	Microbiome diversity	Characteristic shifts in microbial composition	Other notes	Reference
**MTB infection**					
Feces	Murine model study—Comparison of MTB-INH treatment when gut dysbiosis is induced by Abx treatment or not	Total gut bacterial load decreases significantly in Abx-MTB-INH mice compared with MTB-INH mice.	*Lactobacillus* and *Bifidobacterium* decrease, but *Enterococcus* and *Bacteroides* increase in Abx-MTB-INH mice compared with MTB-INH mice.	Antibiotics can impair INH-mediated MTB clearance by suppressing innate and CD4 + T cell responses.	Negi et al. (2020)^109^
Feces	Murine model study—Comparison of RIF vs. INH + PZA pretreated mice challenged with MTB	Compared with untreated mice, RIF treatment induces significant changes in microbial diversity, whereas INH/PYZ treatment leads to modest alterations.	INH/PYZ-treated mice show increased *Bacteroidetes* and *Clostridiaceae* and reduced *Clostridia* IV/XIV compared with untreated controls. RIF-treated mice show increased *Bacteroidetes* and *Verrucomicrobia* and reduced *Firmicutes* and *Lachnospiraceae* compared with untreated controls.	INH + PZA pretreatment increases MTB lung load due to impaired alveolar macrophage activity, but FMT reverses this effect.	Khan et al. (2019)^110^
Feces	Cross-sectional human study—HRZE- treated TB patients and long-term cured patients vs. LTBI controls	HRZE-treated subjects show unchanged Shannon diversity but reduced observed OTUs compared to LTBI controls; cured subjects show increased Shannon diversity with persistent beta diversity shifts compared to LTBI controls.	HRZE therapy causes fecal microbiota dysbiosis (enrichment: *Erysipelatoclostridium, Fusobacterium, Prevotella;* depletion: *Blautia, Lactobacillus, Coprococcus, Ruminococcus, Bifidobacterium*). *Bacteroides* is depleted, but *Faecalibacterium, Eubacterium,* and *Ruminococcus are enriched* in cured patients compared to LTBI controls.	HRZE treatment causes microbiome disruptions that remain detectable for more than a year, indicating a prolonged impact of therapy.	Wipperman et al. (2017)^111^
Sputum	Two clinical trials –PanACEA MAMS-TB and HIGHRIF study 2; retrospective analysis of effect of anti-TB drugs	HR20mg/kgZM and HR35mg/kgZE regimens significantly reduce alpha diversity at weeks 2-8 vs. pre-treatment, while standard HR10mg/kgZE shows no significant reduction	*Neisseria* genus is reduced by 98% in participants whose culture converted by week 8 of treatment. Pre-treatment group is dominated by *Firmicutes* and *Streptococcus*.	Microbiomes recover to baseline by week 12 in most regimens.	Musisi et al. (2023)^113^
Sputum	Prospective cohort study - TB patients (DS-TB vs. DR-TB) vs. healthy controls—Sputum collected at baseline and month 6 post-treatment	TB patients show a lower alpha diversity compared to healthy controls, alpha diversity increases after treatment, more significantly in DS-TB than DR-TB patients.	*Parvimonas* significantly increases after 6 months treatment in both DS-TB and DR-TB, *Megasphaera* increases and *Streptococcus* decreases in DS-TB, *Prevotella* marginally increases in DR-TB.	*Rothia* and *Moraxella* increases are associated with longer sputum-culture conversion time; *Rothia*, and *Gemella* increases are associated with worse pulmonary lesion absorption; Microbiota recovery correlates with favorable treatment outcomes.	Lin et al. (2024)^112^
**NTM infection**					
Feces, Sputum	Prospective longitudinal cohort study—*M. abscessus* PD patients; treatment responders vs nonresponders	Responders show a significant alpha diversity decrease at 2 weeks/6 months treatment compared with baseline (Feces)	Baseline *Eubacterium hallii* higher in nonresponders; 2-week *Enterococcus* markedly increased in responders (Feces)	Responders more frequently use probiotics, implying that supplementation may affect sputum microbiota and support improved outcomes in *M. abscessus* PD.	Kim et al. (2025)^114^
		Alpha diversity decreases in responders only; nonresponders maintain stable diversity. (Sputum)	Baseline *Burkholderia-Caballeronia-Paraburkholderia* and *Porphyromonas* higher in responders; 2-week *Rothia* decreased in responders. (Sputum)		
Sputum	Prospective longitudinal cohort study—NTM-PD patients; serial analysis up to 12 months; subgrouped by culture conversion vs. refractory	Alpha diversity decreases during treatment.	Culture conversion group shows progressive taxa reduction, whereas refractory group exhibits enrichment of *Fusobacterium periodonticum, Veillonella dispar,* and *Pseudomonas aeruginosa.*	Refractory NTM-PD patients may harbor a respiratory microbiota profile that differs from that of treatment-responsive individuals.	Kim et al. (2023)^115^

Source: MTB: *Mycobacterium tuberculosis*; NTM: nontuberculous mycobacteria; TB: tuberculosis; NTM-PD: nontuberculous mycobacterial pulmonary disease; Abx: antibiotics; INH: isoniazid; RIF: rifampicin; PZA: pyrazinamide; HRZE: Standard first-line TB treatment regimen (H = isoniazid, R = rifampin/rifampicin, Z = pyrazinamide, E = ethambutol); LTBI: latent tuberculosis infection; HR20mg/kgZM: Isoniazid (H) + Rifampicin 20 mg/kg (R) + Pyrazinamide (Z) + Moxifloxacin (M); HR10mg/kgZE (standard-of-care): Isoniazid (H) + Rifampicin 10 mg/kg (R) + Pyrazinamide (Z) + Ethambutol (E); HR35mg/kgZE: Isoniazid (H) + Rifampicin 35 mg/kg (R) + Pyrazinamide (Z) + Ethambutol (E); OTU: operation taxonomic unit; DS-TB: drug sensitive tuberculosis; DR-TB: drug resistant tuberculosis; FMT: fecal microbiota transplantation.

Clinical studies indicate that distinct microbiome signatures are associated with treatment outcomes. Standard first-line anti-TB therapy induces long-lasting enrichment of *Erysipelatoclostridium*, *Fusobacterium*, and *Prevotella*, alongside depletion of beneficial taxa such as *Blautia*, *Lactobacillus*, *Coprococcus*, *Ruminococcus*, and *Bifidobacterium* in the fecal microbiota of TB patients. These perturbations can persist for over a year after therapy completion. Notably, in cured TB patients, *Bacteroides* remains depleted, whereas *Faecalibacterium*, *Eubacterium*, and *Ruminococcus* are relatively enriched compared with healthy controls (Table 3).^111^ Extending these observations to the respiratory tract, a multicenter clinical study (HIGHRIF and PanACEA MAMS-TB trials) reported a marked reduction in *Neisseria* abundance among patients who achieved sputum culture conversion after eight weeks of treatment (Table 3).^113^ Complementary findings from another comparative analysis of sputum samples from drug-sensitive (DS-TB) and drug-resistant TB (DR-TB) patients revealed that microbiota diversity recovery is significantly delayed in DR-TB patients.^112^ The dysbiotic microbiota composition characterized by *Moraxella spp., Rothia spp., Parvimonas spp.,* and *Gemella spp*. represents important contributors to pulmonary inflammation, especially in the context of DR-TB infection (Table 3). This differential microbial enrichment suggests that the microbiota composition may function as a critical determinant of TB progression and therapeutic response.

Experimental studies in animal models have demonstrated that microbiome perturbation can compromise the efficacy of anti-TB therapy. Pretreatment with broad-spectrum antibiotics, which disrupts gut microbiota composition, results in increased *Enterococcus* and reduced *Lactobacillus* and *Bifidobacterium* abundance, significantly impairing INH-induced MTB clearance likely through suppression of innate and CD4⁺ T cell responses (Table 3).^109^ Furthermore, anti-TB drugs themselves have been shown to reshape the gut microbiota. Comparative analyzes of RIF and INH plus PZA revealed that both markedly alter the gut microbial composition, with RIF causing pronounced shifts in diversity and INH + PZA exerting milder changes. Following MTB infection, RIF pretreatment has little impact on bacterial burden, whereas INH + PZA significantly increases MTB load, likely due to impaired bactericidal activity of alveolar macrophages. Intriguingly, FMT reverses the elevated MTB burden in INH + PZA-pretreated mice, indicating that specific drug-induced alterations in the gut microbiome can directly influence the efficacy of anti-TB therapy (Table 3).^110^ Taken together, these findings suggest that therapeutic effect on TB is shaped not only by antimycobacterial drug activity but also by the host’s capacity to maintain or reconstitute microbiome-mediated immune competence.

### 4.2. Microbiome signatures associated with treatment response in NTM-PD

As in TB, emerging data suggest that the gut and airway microbiomes also influence treatment responses in NTM-PD.^114^^,^^115^ Our group recently conducted a longitudinal study of *Mycobacterium abscessus* pulmonary disease (*M. abscessus* PD) that revealed significant differences in fecal and sputum microbiome composition between patients with NTM-PD who responded favorably to treatment (‘responders’, defined as culture conversion to negative within two weeks of antibiotic therapy) and those who did not (‘non-responders’).^114^ In this prospective cohort, responders exhibit a marked decrease in fecal and sputum microbial *α*-diversity during antibiotic treatment, whereas non-responders showed minimal change. Baseline enrichment of *Eubacterium hallii* group in fecal samples is associated with poor treatment response, while a pronounced post-treatment increases in *Enterococcus* correlates with favorable outcomes. In the respiratory tract, higher baseline levels of *Burkholderia–Caballeronia–Paraburkholderia* and *Porphyromonas*, together with a reduction in *Rothia* at week two, are linked to favorable treatment response. Interestingly, probiotic use before treatment initiation is associated with responders and coincides with higher baseline abundance of *Burkholderia–Caballeronia–Paraburkholderia*, suggesting a potential interplay between probiotic exposure, microbiome composition, and treatment response (Table 3).^114^

Consistent with these findings, Kim et al. also reported dynamic shifts in the respiratory microbiota during antibiotic therapy for NTM-PD.^115^ Across all patients, sputum *α*-diversity significantly decreases at 1, 3, 6, and 12 months following therapy initiation, reflecting broad microbial suppression. However, the trajectory differs by treatment outcome: in the culture conversion group, most taxa progressively decrease without notable emergence of new taxa, whereas in the refractory cases, *Veillonella dispar*, *Fusobacterium periodonticum*, and *Pseudomonas aeruginosa* increase over time. The enrichment of these taxa in refractory cases suggests that dysbiosis characterized by opportunistic overgrowth may promote microbial persistence and hinder NTM clearance (Table 3).^115^

While human studies to date are largely descriptive, these observations nonetheless suggest that both site-specific dysbiosis and treatment-induced microbial shifts may modulate the host immune milieu and influence treatment outcomes in NTM-PD. The reduction in microbial diversity among responders may reflect the effective suppression of pathogenic or treatment-resistant taxa, whereas the maintenance of diversity in non-responders could indicate microbial persistence under antibiotic pressure, or reflect potential intrinsic differences in antibiotic metabolism or tissue delivery. Microbiome profiling across both respiratory and intestinal compartments thus represents a promising predictive tool for assessing treatment response and relapse risk, and targeted interventions, such as probiotic or nutritional supplementation, may serve as adjunctive management strategies. Unlike TB, where microbiome disruption during therapy is largely driven by drug exposure, microbiome dynamics in NTM-PD appear to reflect intrinsic host–microbial resilience or vulnerability under prolonged antibiotic pressure, potentially explaining the marked heterogeneity in treatment response.

### 4.3. Comparative perspectives on microbiome-mediated treatment outcomes

From a comparative perspective, despite their etiological differences, both TB and NTM-PD exhibit marked dysbiosis at the respiratory and intestinal levels, characterized by a loss of microbial diversity, depletion of SCFA-producing commensals, and enrichment of pro-inflammatory or opportunistic pathobionts. Moreover, accumulating evidence underscores a close association between the microbiome and therapeutic outcomes, while also revealing distinct disease-specific patterns. Alterations in gut and airway microbiota composition have been linked to heterogeneity in treatment responses. Thus, further research is needed to identify the key microbial signatures that are associated with treatment outcomes in TB and NTM-PD and to elucidate the mechanisms underlying these associations. Such efforts may ultimately facilitate the development of microbiome-targeted interventions that could complement conventional antimycobacterial regimens.

## 5. Microbiome-targeted and personalized therapeutics in TB and NTM-PD

Given the growing recognition of associations between the microbiome and treatment outcomes in mycobacterial infections, microbiota-targeted interventions have been proposed to counteract the profound dysbiosis induced by both infection and long-term antibiotic therapy, which contributes to heightened inflammation, immune dysregulation, and poor treatment response (Figure 1). Among microbiota-targeted interventions, the most extensively studied approaches are probiotics, defined as live beneficial microorganisms that, when administered in adequate amounts, confer health benefits to the host (Figure 1. *right*).^116^^,^^117^ In practice, oral administration of live *Lactobacillus plantarum* to antibiotic-treated mice restores anti-TB immunity, concurrent with significant reduction in pulmonary MTB burden compared to the antibiotic-treated controls without *L. plantarum* supplementation.^118^ Similarly, supplementation with *Prevotella copri* to antibiotic-induced dysbiotic mice significantly reduces pulmonary NTM bacterial loads and attenuates lung pathologic inflammation compared with untreated dysbiotic controls. ^27^ One consideration, however, is that the efficacy of probiotics is highly strain-dependent and may be further constrained by host-related factors such as immunosuppression or concurrent antibiotic exposure.^119‐121^ Beyond probiotics, prebiotics, non-digestible food components such as inulin and fructooligosaccharides (FOS), selectively promote the growth and activity of beneficial microbes (Figure 1. *right*).^122^ Prebiotic supplementation enhances microbial diversity and boost SCFA levels, thereby restoring key immunoregulatory functions.^123^^,^^124^ Similar to prebiotics, diets enriched in plant-based fibers have been associated with favorable microbiome profiles and may also reduce gut permeability (Figure 1. *right*).^125^^,^^126^ Because malnutrition and weight loss is both common and associated with poor TB and NTM-PD outcomes, nutritional support represents a particularly critical component of comprehensive care.^127‐129^ Diets rich in fiber, micronutrients, and fermented foods can improve microbiota resilience, enhance immune function, and may synergize with anti-TB therapy.^92,130‐132^ These diet-based interventions are non-invasive and cost-effective. For example, L-arginine, an amino acid derived from diet, endogenous synthesis, and microbial metabolism, decreases in both the serum of NTM-PD patients and NTM-infected mice. The L-arginine supplementation enhances Th1-mediated pulmonary immunity and M1 macrophage polarization, thereby improving bacterial clearance. Moreover, FMT from L-arginine–treated mice confers increased protection against NTM infection, whereas antibiotic pretreatment abrogates these protective effects (Figure 1. *right*).^133^ These results serve as a compelling example of how dietary interventions can remodel the gut microbiota and enhance pulmonary immune defense through GLA.

A more recent and promising approach involves postbiotics, microbial-derived bioactive compounds (e.g., butyrate, propionate, and bacterial peptides) that can directly modulate host immunity. These compounds can be administered without live bacteria and exert anti-inflammatory effects even in the context of severe dysbiosis.^134‐136^ Another comprehensive strategy for microbiota restoration is FMT. FMT, already established for treating recurrent *Clostridioides difficile* infection, has been proposed as a potential therapy for TB and NTM infections.^40^^,^^137^^,^^138^ However, its clinical use remains largely at the preclinical stage owing to safety concerns, regulatory barriers, and substantial inter-donor variability.^139^^,^^140^ Despite these promising preclinical findings, the translation of microbiota-based interventions into clinical practice faces substantial challenges. Differences between animal models and humans in microbial ecology, host immune landscapes, disease stage, and the confounding effects of prolonged multidrug antibiotic exposure severely limit the direct extrapolation of experimental results to TB and NTM-PD patients.

Building on these foundational strategies, future therapeutic directions are shifting toward next-generation microbiome therapeutics, including engineered probiotics, defined microbial consortia, and phage therapy.^140^^,^^141^ These emerging approaches should adopt a comprehensive and personalized framework that integrates patient-specific microbiome profiles, tailored pathogen information, and host immune context. Personalized microbiome profiling can help stratify patients by dysbiosis severity, identify those at risk for treatment failure, and guide optimization of therapeutic regimens. Conversely, clinical observations of microbiome alterations associated with treatment response, disease progression, or immunopathology in TB and NTM-PD should be systematically reverse-translated into experimental systems. The use of gnotobiotic, humanized, or patient-derived microbiota animal models will be essential for establishing causality, defining host–microbe–pathogen interactions, and identifying mechanism-based therapeutic targets.

While still experimental, microbiome-targeted strategies offer a promising adjunct to conventional antimycobacterial therapy and may enhance treatment efficacy and durability through modulation of the host–microbiome interface. Thus, microbiome-based decision-making may become a routine component of TB and NTM management in the future. Ultimately, without rigorous bidirectional validation between clinical cohorts and mechanistic animal models, microbiome-targeted therapeutics will remain largely correlative rather than causally defined.

Finally, integrating microbiome modulation with clinical, immunological, and pathogen-genomic data through multi-omics approaches aided by artificial intelligence will be crucial for developing precision medicine strategies in mycobacterial diseases. Such comprehensive frameworks will enable the design of individualized, evidence-based interventions that translate microbiome science into tangible therapeutic benefits.

## 6. Limitations and future directions toward precision microbiome medicine

Collectively, this review demonstrates that although TB and NTM-PD share common features of airway and gut dysbiosis, their underlying immune–microbiome–pathogen interactions are fundamentally distinct. TB is predominantly characterized by a Th1/Th17-driven, granuloma-centered immunological ecology that favors intracellular containment and latent persistence, whereas NTM-PD arises within far more heterogeneous host environments shaped by structural lung disease, Th2-skewed immunity, and dysbiosis-driven pathogenic inflammatory circuits. Importantly, GLA emerges from this integrated framework not as a passive bystander of pulmonary infection, but as an active immunoregulatory network that differentially shapes host susceptibility, disease progression, and treatment responsiveness in TB and NTM-PD (Figure 1).

These insights reposition the microbiome from a purely correlative biomarker to a mechanistically relevant modulator of mycobacterial disease phenotypes and a rational target for host-directed and personalized therapeutic strategies. Microbiome perturbations appear to participate in a bidirectional feedback loop with mycobacterial infection, in which pulmonary infection reshapes gut microbial communities, while gut-derived metabolites and immune signals, in turn, readjust the state of pulmonary immune system. Within this framework, microbiome-based precision medicine represents not simply an adjunctive treatment, but a potential means of reprogramming host–pathogen equilibrium.

Nonetheless, several important limitations should be acknowledged. First, most available studies are cross-sectional, making it difficult to establish temporal or causal relationships between microbiome alterations and disease outcomes. Second, substantial methodological heterogeneity, including differences in sample types (sputum, bronchoalveolar lavage, or stool), sequencing platforms, and bioinformatic pipelines, complicates cross-study comparability and highlights the urgent need for harmonized analytical frameworks to ensure reproducibility and reliable meta-analyzes. Third, critical host-related confounders such as prior antibiotic exposure, nutritional status, and underlying comorbidities are often incompletely controlled, thereby limiting causal inference. Finally, there remains a critical lack of parallel, head-to-head studies analyzing the microbiome of TB and NTM-PD patients using standardized methodologies, which constrains our ability to define disease-specific microbial signatures and shared versus divergent host–microbe interaction pathways.

In addition to these conceptual limitations, methodological challenges inherent to airway microbiome profiling warrant careful consideration. Because the lower respiratory tract harbors a low-biomass microbial community, sequencing-based analyzes are highly susceptible to contamination from the upper airway, bronchoscopy channel, environmental sources, and laboratory reagents. Indeed, a substantial proportion of taxa detected in BALF or protected specimen brushing samples may originate from oral carryover or reagent-derived background signals rather than representing true lower-airway residents.^142‐144^ The lack of rigorous negative controls, including bronchoscopy wash controls and extraction blanks, further complicates the distinction between genuine pulmonary microbiota and technical artifacts.^78^^,^^88^^,^^94^ These issues underscore the necessity for strict environmental controls, standardized sampling protocols, and conservative analytic pipelines when interpreting airway microbiome data in TB and NTM-PD. Without such safeguards, apparent disease-associated microbial signatures may, at least in part, reflect contamination rather than biologically meaningful ecological shifts.

Beyond these technical sources of noise, a fundamental unresolved question concerns the intrinsic stability of the lower airway microbiome under baseline, non-diseased conditions. Even in healthy individuals, the pulmonary microbiome is thought to exhibit low-level stochastic fluctuations driven by microaspiration, normal mucociliary clearance, and the transient movement of microbes from the upper airway.^145^^,^^146^ Moreover, the overall immune status of the host, ambient environmental exposures such as air pollution and smoking, systemic factors such as diet and metabolic health may all contribute to differences between individuals and changes over time.^147‐149^ This inherent ecological volatility complicates the definition of a universal ‘healthy’ lung microbiome and requires careful consideration when interpreting disease-associated dysbiosis in TB and NTM-PD.

Future research should prioritize longitudinal cohort studies capturing microbiome dynamics before, during, and after therapy, ideally integrated with synchronized immunologic and metabolic profiling. Comparative meta-analyzes of TB and NTM microbiomes will be essential for distinguishing conserved dysbiosis patterns from disease-specific microbial trajectories. Moreover, integrating multi-omics platforms, including microbiome, transcriptomic, immunologic, and metabolic profiling, will provide a systems-level framework for dissecting GLA-mediated mechanisms in mycobacterial disease.

Ultimately, microbiome-targeted interventions may serve as valuable adjuncts to conventional anti-mycobacterial therapies, offering the possibility of restoring immune homeostasis, enhancing treatment durability, and reducing relapse risk. The convergence of microbiome science with artificial intelligence–assisted multi-omics integration holds particular promise for translating complex microbial and immunological signatures into actionable clinical decision-making tools. Such precision medicine frameworks will be essential for transforming microbiome research from associative discovery into individualized, mechanism-based therapeutic strategies for both TB and NTM-PD.
